# A Memoir of Inventing Real-Time PCR and Developing the ABI 7700

**DOI:** 10.3390/ijms27062612

**Published:** 2026-03-12

**Authors:** Russell Higuchi, Lincoln McBride

**Affiliations:** 1 Independent Researcher, Alameda, CA 94501, USA; 2 Independent Researcher, Belmont, CA 94002, USA

**Keywords:** real-time PCR, analytical instrumentation, DNA synthesizer, *Taq* polymerase, phosphoramidite, HIV, COVID-19 testing, fluorescent probes, Cetus, Applied Biosystems, Roche

## Abstract

Real-time PCR (qPCR) is today’s definitive quantitative technology in molecular biology and diagnostics. Until 30 years ago, PCR product analyses were generally performed after amplification using gel-based methods. Quantification typically relied on visual inspection or densitometry of end-point products and was therefore relatively unreliable and poorly suited to high-throughput automation. To celebrate real-time PCR’s 30-year anniversary of commercial availability, Professor Stephen Bustin, Guest Editor for the special edition, “Advancing Molecular Science Through Reproducible qPCR: MIQE Guidelines and Beyond,” asked Russell Higuchi to give a historical account on how his idea of real-time PCR was conceived and brought to fruition. Dr. Higuchi then asked his collaborator, Lincoln McBride, who drove the development of the ABI 7700—the high-throughput real-time PCR instrument that gave researchers access to this technology—to co-author this dual memoir. This story is told from the perspectives of the two scientists most directly responsible for making real-time PCR practical and widely accessible. Taking turns, Russell Higuchi describes the conceptual and experimental steps at Cetus and then Roche that led from homogeneous PCR detection to continuous fluorescence monitoring, whilst Lincoln McBride details ABI’s parallel efforts to commercialize Russ’s invention. Together, they trace how experimental insight, engineering constraints, product development, and commercial decision-making shaped the Applied Biosystems 7700 Sequence Detection System and established real-time PCR as a practical and reliable quantitative technology. Their team’s efforts persevered through technological uncertainty and within a complex corporate collaboration. They share key historical documents in their original form. Their accounts show how the 7700 system emerged as the convergence of chemistry, optics, software, and product development. The eventual global reliance on real-time PCR during the COVID-19 pandemic demonstrated, at unprecedented scale, the profound and enduring impact of these early technical and organizational choices.

## Russ Higuchi

The project that became “real-time PCR” began in November 1986 after I joined Cetus—then a major company in the recombinant DNA-fueled biotech industry and where Kary Mullis had conceived of PCR in 1983. By 1986, David Gelfand’s group at Cetus had cloned, characterized, and expressed a thermostable polymerase, *Taq*. Because *Taq* survived the high-temperature denaturation phase, PCR could be done in an unopened tube. Tom White, who hired me, said the next step for full automation was to do both the amplification and detection of amplicon in an unopened tube. We needed to find a way for the amplicon to signal its own generation. This “homogeneous” PCR detection would not only be amenable to automation, but a closed tube should also prevent the amplicon from contaminating the workspace, thereby minimizing false positives during subsequent PCRs [1].

Cetus was my first job after my postdoc at Berkeley in the Biochemistry Department. Before this, I had interviewed in academia and interestingly enough at Applied Biosystems (ABI). But my postdoc advisor, Allan Wilson, had arranged a project for me at Cetus, where his former students, Tom White and Norm Arnheim, were using an amazing new method, the “Polymerase Chain Reaction,” invented by another former Berkeley Biochemistry student, Kary Mullis.

In Allan’s lab, I used conventional molecular cloning methods to isolate and sequence the first bit of DNA from the African Quagga [2], an extinct species (or as the result showed, a subspecies). Allan coined the term “Ancient DNA” from this work—his postdoc researcher, Svante Paabo, would in 2022 earn a Nobel Prize for using next-generation sequencing (NGS) on entire ancient genomes. Before NGS, we were happy to get just a little bit of DNA sequence information and PCR promised to greatly facilitate this.

After learning PCR in Henry Erlich’s group at Cetus, I applied it to other ancient samples like frozen tissue from a wooly mammoth. I really liked Cetus and applied for a job in Henry’s group. My interview seminar was titled “DNA of the Dead” and happened to coincide with Halloween. I was hired and started working on DNA sequencing and forensic science applications of PCR. In 1988, we published our first paper using PCR for human identification from a single hair [3]. Henry Erlich has written a book on the application of PCR to forensic science and human genetics [4].

By 1989, I was ready to meet Tom White’s challenge. Gavin Dollinger at Cetus was a physicist and specialist in analyzing proteins by light scattering. He said that if we could turn amplicon into massive aggregates or multimers, we could use light scattering to detect them in the closed PCR tube. I suggested adding biotin to the ends of the PCR primers and including streptavidin in the PCR mix. The idea was that tetravalent streptavidin would bind to biotin, creating multimers once PCR got going. However, when we tried this, flocculent precipitates formed in all PCRs, whether samples contained target sequences or not.

I asked Bob Griffith, whom I supervised, to add ethidium bromide (EtBr) to completed streptavidin PCRs [5]. The fluorescence of EtBr increases when it intercalates into double-stranded DNA, e.g., PCR amplicon. We used a handheld UV light to see if the precipitates fluoresced, an indication that amplicons were participating in multimer formation. To my surprise, Bob had added EtBr to the PCRs before—not after—the thermal cycling. I had assumed that EtBr, an inhibitor of DNA polymerase, inhibited PCR. Bob also ran a control PCR containing EtBr but lacking streptavidin, which surprisingly showed a DNA band of correct size by agarose gel electrophoresis. Apparently, our concentration of EtBr was below inhibitory levels. We took this control PCR tube to the darkroom and held it over a UV transilluminator. It glowed strongly!

I was eager to see if we could detect increasing fluorescence in “real-time” during thermal cycling.

**Figure 1:**
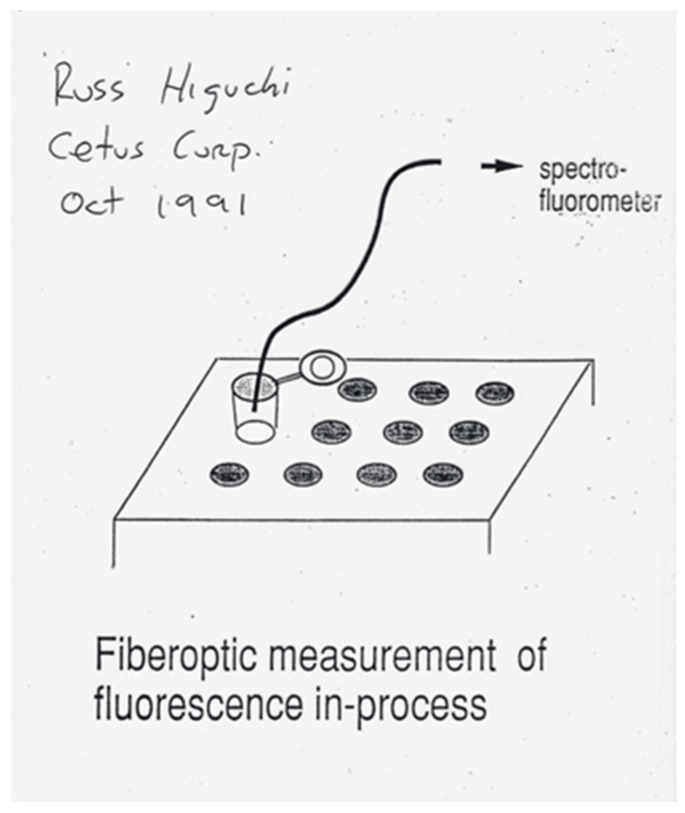
First public disclosure of the real-time PCR system. Russ Higuchi’s overhead transparency presented at the IBEX Conference by Russ Higuchi on 9 October 1991, at Brooks Hall in San Francisco, CA, and then given to Lincoln McBride to present at ABI.

I asked Gavin if he could connect a fiber-optic cable from the PCR tube to his spectrofluorometer. He ordered a special cable attachment and, when it arrived, he used optical grade epoxy to glue one end of the cable to the top of the PCR tube. He connected its other end to the light source in the spectrofluorometer. Through this bifurcated cable, excitation light was sent to the PCR and emitted light was sent back to the spectrofluorometer for analysis (Figure 1).

**Figure 2:**
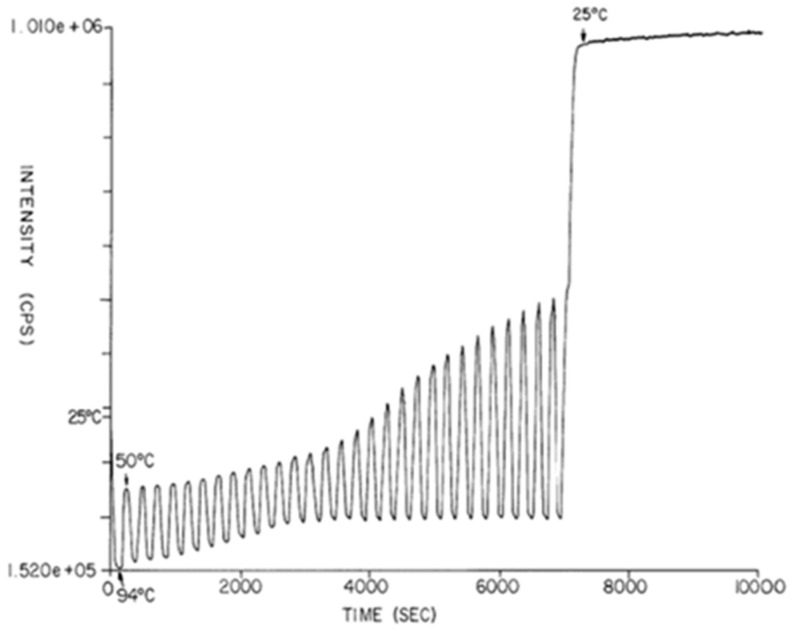
Continuous fluorescence monitoring of a PCR. A PCR containing EtBr is monitored using a bifurcated fiber-optic cable as in Figure 1. Fluorescence intensity is on the *y*-axis and time is on the *x*-axis. The down-and-up in fluorescence corresponds to PCR thermal cycling from 94 °C (denaturation) and 50 °C (annealing and extension) for 30 cycles. The amplification was using human male DNA-specific primers in a PCR starting with 20 ng of human male DNA.

When the first PCRs were read this way, we observed emission light from EtBr throughout the full range of thermal cycling temperatures. A plot of fluorescence intensity as a function of time resulted in a down and up, sinusoid-like pattern (Figure 2) [6]. The known inverse dependence of fluorescence intensity upon temperature was reducing fluorescence during denaturation and increasing it during primer extension. This down and up of fluorescence intensity stayed within a relatively constant range during early cycles. In later cycles, as amplicon accumulated, the extension phase fluorescence incrementally increased, eventually reaching a plateau. Today’s systems use fluorescence only during the extension phase to trace a function referred to as a PCR growth curve. Based on these results, we filed the first real-time PCR patent in May of 1991 [6].

But just two months later, we learned that Cetus would soon cease to exist. The clinical trial of its lead drug, Interleukin-2, had not met its endpoint and Cetus could not survive without the drug’s approval. The newer and neighboring company, Chiron, took over Cetus and its pharmaceutical assets. However, patent rights to the most valuable application of PCR, in vitro human diagnostics, were sold to Hoffman-La Roche for $300 million. The PCR rights for all the other markets remained with Perkin-Elmer (PE), who, in joint development with Cetus, had commercialized the first thermal cycler and PCR reagents. These changes in patent rights would soon prove quite significant for ABI, as Lincoln describes later.

For another six months, our job status remained in limbo because Cetus’s merger with Chiron still required SEC and stockholder approval. It was at this time that I gave a seminar at IBEX, the International Biotechnology Expo, in San Francisco. I felt it would not hurt to put my name and work out there in front of potential new employers. Since our patent had been filed, I asked for permission to present this nascent real-time PCR work at IBEX, and it was granted.

## Lincoln McBride

Was it an omen? IBEX mistakenly listed me as a speaker from Cetus. As Applied Biosystems’s (ABI) scientific spokesman, I had given this talk, “The Importance of the Polymerase Chain Reaction in Automated Genetic Analysis,” seemingly non-stop for two years. Russell Higuchi, who actually did work for Cetus, spoke right after me. During his presentation, “Applications & Analytical Instrumentation—Current & Future,” he disclosed his invention for monitoring a PCR in real-time using the fluorescence of a DNA intercalator. Russ’s analytical device was a perfect fit for ABI. As he finished, I hurried to the stage to say we were experts in fluorescence-based DNA analysis and could help commercialize his instrument. Russ was clearly interested.

The morning after IBEX, I found Bob Jones, ABI’s VP of R&D. Never before had I approached management about an idea I had seen on the road. ABI’s pipeline of new product lines was thin, and Russ’s system would leverage ABI’s extensive fluorescence technology developed for our DNA Sequencer. ABI’s DNA sequencing business was still lackluster, so it was appealing to leverage its technology. After describing Russ’s clever invention, I said Russ was receptive to us building it. Bob gave me the green light to start an investigation and would coordinate with Cetus’s management.

Bob had me present Russ’s invention at ABI’s “New Ideas Day.” Russ supplied me with his overhead transparency showing an optical fiber coming out of a PCR tube (Figure 1). I emphasized this product could propel ABI into diagnostics, a more sustainable business than research tools. Alex Andrus, the New Ideas Day lead judge, announced the winners. After a final long pause, Alex said, “We felt we need an additional award, but don’t know what to name it…it goes to Lincoln.”

Immediately, I became real-time PCR’s program leader—a job that would last for six whirlwind years. My internship as an analytical chemist at the Upjohn Company [7] and my DNA synthesis expertise gave me confidence this technology would be a winner. Real-time PCR requires deoxyoligonucleotide (“oligo”) primers and fluorescent probes, both made with DNA synthesizers. In 1980, I joined Marvin Caruthers’ lab as a graduate student in organic chemistry at the University of Colorado in Boulder. I chose Boulder because it was my birthplace and chose Marv’s lab because it was the birthplace of DNA synthesizers [8].

Months before my joining, Marv’s group revolutionized DNA synthesis and therefore biotechnology [9]. Graduate student Mark Matteucci invented solid-supported DNA syntheses on silica. Then came the pivotal discovery of the phosphoramidite method by postdoc researcher Serge Beaucage. Oligos made with these technologies enabled incredible innovations, including whole gene synthesis, gene regulation and editing, DNA fingerprinting, the first human genome sequence, and PCR.

My Ph.D. thesis, “Improvements in the Chemical Synthesis of Deoxyoligonucleotides” produced a few modest structural and process improvements in phosphoramidite synthesis and was work I had a knack for. I lacked curiosity and patience for open-ended research, preferring building more than publishing. It is also noteworthy that Marv implanted in me the idea that developing a highly sensitive, non-radioactive DNA probe would be challenging but valuable for research and diagnostics. Bill Efcavitch and Curt Becker had left Marv’s lab to help ABI commercialize their 380A DNA synthesizer. Developing new instrument-reagent systems for ABI seemed a perfect fit for me. In 1985, I became ABI employee 258.

While exiting New Ideas Day, several colleagues volunteered to develop Russ’s idea. The next day we started working on real-time PCR! Martin Johnson, Kip Connell, Linda Lee, and John Shigeura cobbled together our first real-time apparatus. Marty machined a custom PCR tube cap from polypentene. It was indented so an optical fiber fit snugly. We illuminated the other fiber end with an $8000 argon ion laser and measured fluorescence emissions traveling through the same fiber in the opposite direction with a photomultiplier tube taken from a 370A DNA sequencer [10]. Kip was the optical engineer on that pioneering instrument. Initially, we used ethidium bromide (EtBr) and detected fluorescence continuously for several seconds during each extension phase. We aimed to witness a signature PCR growth curve on a semi-log plot of Log (fluorescence intensity) versus cycle number. We expected to see a linear section while the amplicon concentration was doubling each cycle, called the log phase, followed by a section that gradually became horizontal as the PCR replication efficiency decayed.

**Figure 3:**
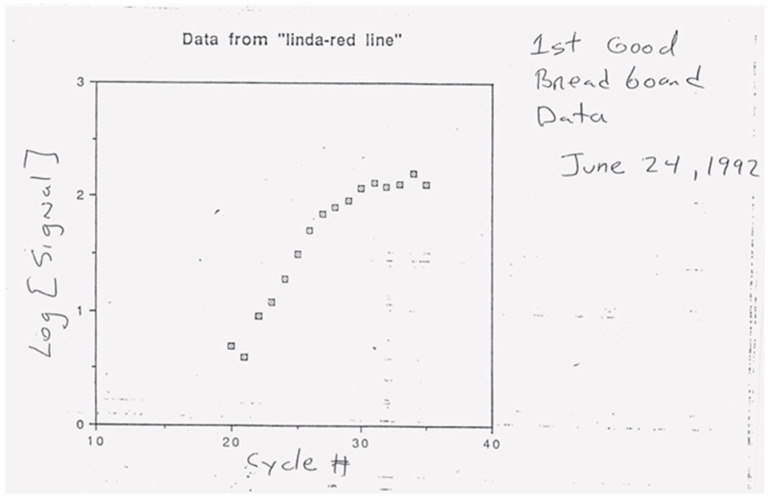
First detection of a PCR signature. ABI’s first generated real-time PCR-like signature using ethidium bromide. A plot of Log (fluorescence intensity) versus cycle number. This data was from the single sample, single fiber, real-time PCR apparatus with fluorescence hardware from an ABI DNA sequencer using its 514 nM excitation and 610 nM emission filters [10]. The image is from a 1992 overhead transparency.

On 24 June 1992, we generated our first PCR signature, six cycles in PCR’s log phase (Figure 3). Common sense and intuition said that duplicate PCRs were the most uniform tube to tube in their log phase. So, I set the product’s key requirement to detect PCR for at least three cycles in the log phase. Other requirements included quantifying target amounts over a dynamic range of 1 million and a parallel capacity of 96 PCRs in a microtiter plate-like format.

## Russ Higuchi

The 1991–1992 transition from Cetus to Roche was an interesting time. Making it even more interesting, that November my wife and I welcomed our first child into this world!

We knew there should be a quantitative aspect to comparing PCR growth curves from differing amounts of starting target sequence. The more target sequence to begin with, the fewer cycles it should take to first detect fluorescence. Knowing how precisely quantitative this could be was extremely important for the then most important clinical application of PCR: HIV viral load measurement. However, a single fiber-optic cabe crudely attached to a single tube run in isolation made it difficult to determine PCR’s precision. So, Gavin ordered a “multiplexing” fiber-optic cable system usable with his spectrofluorometer. Perhaps a dozen PCRs could be run in parallel. However, it would require six months to custom fabricate this cable system.

Meanwhile, the sale of Cetus PCR assets had finalized. Our jobs would transfer to Roche, but we would lose our stock options. Cetus executives, on the other hand, had massive “golden parachutes,” allowing them to exercise huge stock options. This did not sit well with us scientists who had innovated and fought hard to establish PCR as a viable technology, despite frequent resistance from these same executives who had prioritized the failed pharmaceutical projects. So, we went on strike! I was one of the leaders that helped build a consensus among the scientists. We would refuse to sign contracts with Roche unless they reinstated our options and gave us decent raises. After many very stressful meetings and strained personal relationships, we finally got a small raise, our options reinstated, and became Roche’s PCR group.

Focusing on work again, I wrote our first paper describing homogeneous detection and real-time PCR [11]. The optical multiplexer still had not arrived, so I started playing with the then new, open source “NIH Image” software [12]. It was a revelation that it allowed one to highlight regions of interest in a digital image and sum the pixel values within them. I knew that Bob Watson, in David Gelfand’s group, had assembled a photo stand with a digital camera for photographing EtBr-stained gels on a UV transilluminator. Digital cameras in the early 90s were expensive! Bob’s low noise 8-bit camera cost $10,000. I asked Bob if we could aim his camera at the tops of PCR tubes containing reactions with EtBr—bypassing the need for fiber-optic connectors. The tubes would sit in the heating/cooling block of a PE-Cetus 480, the first commercially available thermal cycler. Could we take digital images of the tubes in the block under fluorescence illumination? And could regions of interest in these images corresponding to solutions within the tubes (as seen from the top) be analyzed? And could all this be done on a cycle-by-cycle basis during the the extension phase of PCR to trace real-time growth curves?

**Figure 4:**
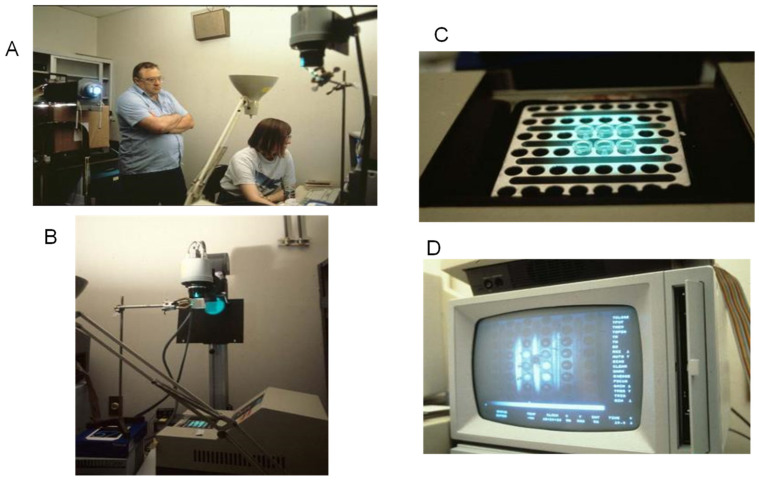
Array-based imaging of real-time PCR. (**A**) Bob Watson and Carita Fockler in 1992 in a darkroom together with a digital camera pointing at the heating/cooling block of a PE 480 thermal cycler with open PCR tubes, capped by mineral oil to prevent evaporation. (**B**) The projector aims light filtered to 500 nM at a dichroic mirror that reflects it toward the thermal cycler block. Emitted > 600 nM light passes through the dichroic mirror to the camera. (**C**) Close up of an illuminated area of the block. (**D**) Image capture by camera showing the tops of the tubes.

The answers were all yes. As described in [13], the camera was on a stand pointing down at the thermal cycler block in a darkroom (Figure 4). Illumination was provided by filtered 500 nm light from a 1000-watt tungsten lamp. The caps were removed from the tubes but evaporation was prevented by a layer of mineral oil. The camera was hoisted as high as possible above the thermal cycler to prevent parallax from blocking the view of the reactions at the bottoms of the tubes. Excitation light was reflected to the reaction block by a dichroic mirror that allowed longer wavelength emission to pass through it to the camera. At first, we had to sit in the darkroom during the entire PCR to trigger the camera manually during the extension phase. The computer controlling the camera stored the images for later analysis of regions of interest corresponding to each tube’s top.

**Figure 5:**
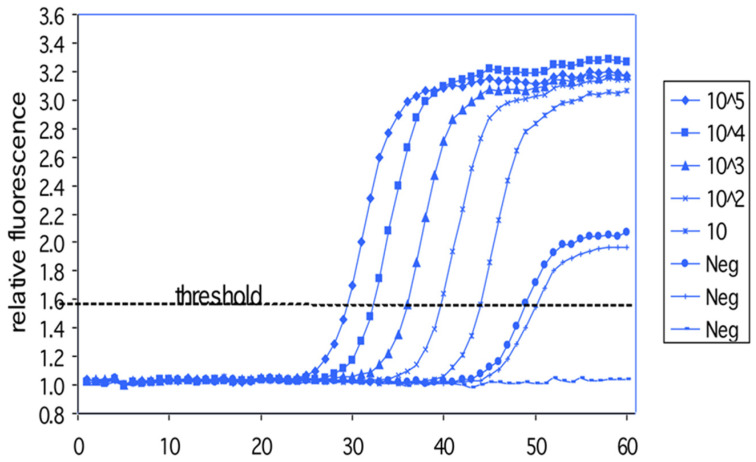
PCR growth curves from a 10-fold dilution series of target DNA. Growth curves (normalized fluorescence vs. PCR cycle number) in response to a 10-fold dilution series of template DNA (HIV sequences in a plasmid). These PCRs were monitored on the setup in the previous figure. The log starting copy numbers of template DNA in a 100 microliter PCR is shown in the legend.

Now we had space for as many as 48 replicate PCRs to assess the reproducibility of real-time PCR. Analysis of a 10-fold dilution series of target sequences using the data normalization scheme described in [13], gave a nearly identical set of growth curves but offset by about 3.3 cycles between dilutions (Figure 5; note that these are linear plots of fluorescence as opposed to the semi-log plot in Figure 3). Such a set of curves is still used today for the calibration of quantitation. It is so emblematic of real-time PCR that stylized versions have been used as logos of PCR-based companies—PCR Biosystem, for one. However, ours were the first such curves ever seen.

When I printed out the set of curves, I took them into the hallway looking for someone with whom to share my excitement. I ran into John Sninsky, who had led Discovery Research of the PCR division at Cetus. John was the first to recognize the value of PCR in monitoring the HIV virus in AIDS patients [14].

Conventional wisdom was that PCR could only be semi-quantitative at best; small tube-to-tube variations in replication rate would be exponentially amplified, resulting in wildly different trajectories of growth in replicate PCRs. My data indicated this was not the case, but I did not understand why. John had already thought about it and was not surprised. He explained, the “stop and start” of DNA replication during PCR was like synchronized cell culture where, because of cell-cycle blockers like colchicine, cells are blocked from replicating more than once until the colchicine is briefly washed out, and then reapplied. In PCR, provided that sufficient time was allowed and the reactants were not limiting, every cycle should double—but not more than double—the amplicon concentration. This should be true throughout the log phase. In addition, particularly if the starting DNA concentration is high enough, synchronization persists into PCR’s decay phase as the increases become linear rather than exponential. It is when non-specific PCR products occur with low inputs of target DNA that non-identical curves often result.

Determining the fractional cycle number needed to reach a set threshold level of fluorescence, later termed threshold cycle (Ct) by ABI, gave a precise measure of the starting copy number of DNA molecules. This feature, together with the six-log dynamic range real-time monitoring provided, would enable accurate HIV viral load measurement.

The quantitative value of PCR’s exquisite synchronization is clear upon comparison with isothermal methods for amplification. Without the rate-limiting effects of thermal cycling, isothermal reactions have kinetic characteristics expected from exponential processes. In Hermann et al. [15], Ct for PCR is log-linearly related to the starting copy number while, on the same samples, time-to-detection by the isothermal method RHAM, a variant of LAMP, is hardly related to the starting copy number at all, even though viral positivity correlates with an increase in RHAM fluorescence.

## Lincoln McBride

On 6 October 1992, I phoned Russ from Tokyo with incredible breaking news. ABI would merge with PE, giving us license to commercialize real-time PCR. In February, Bob Jones became PCR Business Unit Manager of the PE Applied Biosystems division of PE in Foster City, CA. I remained real-time PCR’s program leader for both R&D and marketing. Bob Ragusa, a PE engineering leader, would lead PCR R&D, becoming my sixth boss in as many years. Although a man of few words, Bob shared this wisdom: like a dog with a bone, hold on tightly to the product’s key analytical requirement.

Because EtBr detects all double-stranded DNA, my fear was new assays would require optimization and validation with gel electrophoresis. Worse, false positives could cripple the reputation of real-time PCR. So, we turned to DNA probes labeled with fluorophores. David Gelfand’s group at Cetus had demonstrated that *Taq*’s 5’-nuclease activity could make a DNA probe “read-out” during PCR [16]. Although their assay used electrophoresis and autoradiography, the last sentence of their paper became our future: “…we envision the use of this technique to create a truly homogeneous assay, in which amplification and actual detection occur in one tube.”

ABI was the leader in labeling oligos with fluorophores. Our collaborator, Jerry Ruth, said that in a probe with two fluorophores, one could quench the other [17]. As Holland et al. [16] hinted, the 5′ nuclease of *Taq* polymerase might separate fluorophores on an annealed probe, boosting fluorescence. Because we had an extensive toolbox of modified phosphoramidites, fluorophores, linker arms, and abundant oligo sequence flexibility, I was highly confident we could succeed in engineering a fluorescent probe motif. Mel Kronick, our father figure in ABI’s fledgling DNA diagnostics effort, suggested this first experiment. I synthesized an oligo with multiple fluoresceins and subjected it to snake venom phosphodiesterase in a fluorimeter to see if it both digested and gave an increased fluorescent readout. Fluorophores in close proximity typically self-quench. After 30 min of nuclease exposure, there was a five-fold increase in fluorescence.

Linda Lee, ABI’s dye chemist, started designing fluorescent “TaqMan” probes. Using dyes she helped develop for sequencing, she labeled an oligo at its 5′-end with fluorescein (FAM) and placed a rhodamine (TMR) seven nucleotides from FAM. She chose TMR as the “quencher” since its excitation spectrum overlaps the 520 nm emission of the “reporter,” FAM. This double-labeled, sequence-specific probe worked well in real-time PCR [17], which was incredibly encouraging.

However, only approximately half of our probes designed this way were sufficiently quenched, so more work was needed to find a universal probe design motif. Back then, we were not certain TaqMan probes could fulfill our requirements. This 1994 summary quote—from our Specifications & Features Description, Rev. 6.5, 28 May 1994—describes our flexible design philosophy: “Because the analytical thermal cycler (ATC) is a first-generation analytical technology, our understanding of which specifications will be most important to mainstream customers is incomplete. Therefore, design decisions that we make may be biased toward greater flexibility, higher performance, lower running cost and quicker time to market, possibly at the expense of higher instrument cost.”

In this spirit, we future-proofed the 7700 with high-sensitivity hardware and flexible spectral detection to adapt to emerging needs. By providing access to raw fluorescence data, we enabled expert users to experiment with new applications. Although TaqMan became the standard, the 7700 also proved effective with alternative chemistries [18].

In response to severe R&D mistakes during development of the 370A DNA sequencer and the 380B DNA synthesizer, ABI was moving to an ISO 9001 [19] project management system. My boss, Elaine Heron, encouraged me to have the 7700 be the first major new ABI product line to adopt ISO 9001. It became an invaluable roadmap for me. Jan Hughes, who helped draft our ISO guidelines, recommended I identify critical technical issues and demonstrate feasible solutions independently for each. Among 14 critical issues, my main concerns were having the sensitivity to detect three cycles in the log phase on 96 samples and identifying a practical TaqMan probe synthesis strategy that works for all genetic targets.

Tim Woudenberg, our systems engineer, explained our fluorescence sensitivity challenge: to reliably detect the faint emission light resulting from probe cleavage. This light was “swimming” in a pool of excess fluorescence generated from an intact TaqMan probe. Far more probe was present than would ever be cleaved and intact probes were at best 90–95% quenched. We estimated PCR’s log phase ended when amplicon concentration reached 1 × 10^−8^ mol/L. So, detecting three cycles in the log phase meant realiably detecting an increase in fluorescein corresponding to 1 × 10^−9^ mol/L in the presence of an effective fluorescein concentration as much as 20-fold higher. Tim calculated that analyzing 96 wells simultaneously for several seconds with flood illumination would not be sensitive enough. However, by radiating 96 tubes one at a time for 50 milliseconds each, an argon ion laser should work!

**Figure 6:**
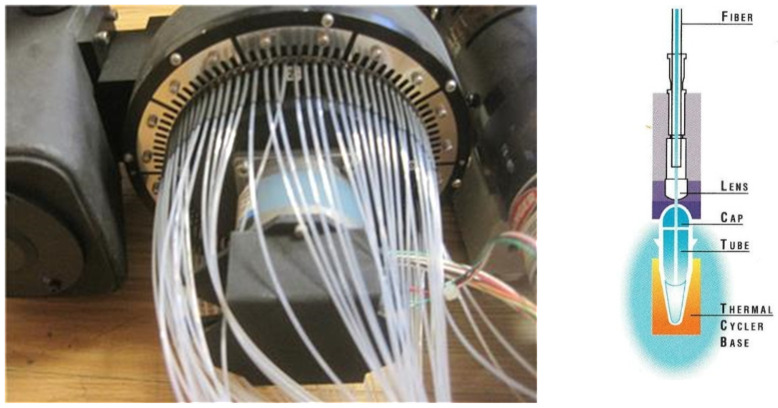
(**Left**) Image of the multiplexer with fibers attached. This MUX is attached to the 7700’s optical block with the optical fibers attached around its circumference. (**Right**) Close up of the 7700 optics. This figure shows the positioning of the optics above each PCR tube and well of the thermal cycler block. The figure is from a 1996 ABI sales brochure.

Our challenge became developing a rapid scanner with a highly stable and reproducible optical path to minimize noise in a high fluorescence background. Eugene (Gene) Young, PE’s senior engineer, designed our scanner, calling it a multiplexer (MUX). Its tiny enclosed periscope revolved 360 degrees every eight seconds like the ticking second hand of a clock. Optical fibers were attached around the MUX’s circumference (Figure 6, Left). The periscope’s outer end delivered laser light to all 96 optical fibers one by one, with a dwell time of 50 milliseconds each. The other ends of the fibers were positioned closely above each corresponding PCR tube (Figure 6, Right). Both 488 nm laser light and fluorescence emission light at 500–660 nm traveled through the fibers, but they traveled in opposite directions as diagramed by Woudenberg et al. in their second figure [20].

A dichroic mirror let laser light pass into the PCR solution but reflected the returning emission light towards the CCD detector. My feedback to Gene was that the MUX’s optical stability appeared risky. Gene politely chuckled, surely knowing otherwise. He would prove that PE optical engineering was world class. Gene’s MUX is now in the Smithsonian Institute, National Museum of American History (ID 2023.0023.01).

As insurance against excessive optical instability, we added a passive fluorescence reference standard (ROX) to each PCR [21]. All fluorescence measurements in the FAM channel were divided by their corresponding measurements from the ROX channel. This ratio (R) helped cancel noise in the fluorescence baseline caused by unwanted optical path fluctuations from possible mechanical vibration or imprecision, and possible movements of polypropylene caps and tubes, bubbles, menisci, or droplets. ROX helped ensure adequate and reliable sensitivity in the log phase by enabling the 7700’s threshold to be set closer to the growth curve’s baseline.

## Russ Higuchi

Our quantitative results continued to improve. We now demonstrated precise quantitative detection of HIV DNA sequence standards at 100 copies. Equally gratifying, Bob freed us from babysitting the setup in the darkroom by rigging a trigger from the printer port on the thermal cycler to our digital camera. The port sent text to the camera once the extension temperature was reached. UV lamps replaced the tungsten light source and illumination was spread to encompass most of the thermal cycler block. To read out quantitative results from growth curves, Bob adapted a gel analysis program to store the camera’s pixel values corresponding to each PCR tube top. These images were taken at the end of every PCR extension phase and stored for post-PCR analyses.

This data was entered into a spreadsheet I created, which did both cycle-to-cycle and well-to-well normalization of fluorescence values. Cycle-to-cycle normalization required a dedicated well with a PCR tube containing a baseline level of EtBr and primers, but no template or polymerase. Its inherent fluorescence was not expected to vary during a run. The fluorescence variations observed in each cycle from this control tube were used to normalize the fluorescence values from the other 47 tubes by division. Well-to-well normalization used an initial fluorescence measurement as a divisor for all subsequent measurements from that well.

Another feature of the spreadsheet was the determination of the intercept (Ct) between the fluorescence threshold and the growth curve. Although the threshold could be determined algorithmically, in practice, we manually set it as low you could go without intercepting noise in the baseline phase of the growth curve. My spreadsheet calculated the intercept using an interpolated log line to the fluorescence for three cycles above the threshold and for two cycles below. I sent a version of the analysis spreadsheet to ABI. Similar features were incorporated into the analysis software for the 7700 and for all real-time PCR instruments since.

Lastly, I added a feature I came up with that calculated the initial amplification efficiency, which optimally should be 100% of a full doubling [4]. It uses the slope of the calibration curve taken from the Ct values of a dilution series of targets at different concentrations—usually 10-fold dilutions. The difference (slope) between these Ct values in a 10-fold dilution series should be about 3.3 cycles if the efficiency is 100%, as expressed in the equation (10^−1/slope^ − 1) × 100% = per-cycle efficiency [5]. This method of efficiency calculation is part of the MIQE guidelines [22].

## Lincoln McBride

A year had passed since the merger and we were knee deep in our commitment financially, professionally, and personally to make real-time PCR a success. Tim constructed a real-time PCR prototype fitted with four optical fibers attached to Gene’s MUX. It took three runs to analyze all nine DNA standards spanning one thousand to one million β-actin gene copies. We seemed on the verge of generating our first solid proof of principal data. However, our first standard curves had unsatisfactory R-squared values. Tim did not seem fond of my hovering during those suspenseful months.

Paul Wyatt was our team leader from chemical manufacturing. One morning, Paul approached me with his hypothesis that the poor standard curves were caused by inaccuracies in the standards themselves. He believed Tim’s prototype was detecting indeterminate errors from our formulation procedure. Paul wanted my approval to prepare new standards unconventionally. He would formulate the most concentrated standard and serially dilute it with volumetric flasks to make the others. These standards were not more accurate but were internally more consistent.

**Figure 7:**
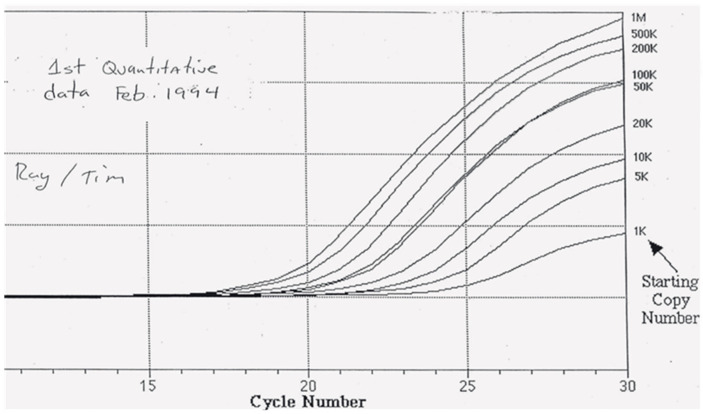
Proof of principal data of 7700 architecture for probe-based real-time PCR. Real-time amplification plots from February 1994 using a TaqMan probe and PCR primers for the β-actin gene. A set of standards from 1 K to 1 M copies of β-actin DNA were analyzed after combining three runs from a real-time PCR-MUX prototype fitted with four optical fibers. Image from a 1994 overhead transparency.

About a week later, Ray Lefebvre, my software and algorithms R&D leader dropped by my desk. Up until this point, Ray did all our coding and data analysis. Eventually, he hired an extremely close-knit team, Jeff Lucas, Bruce Goldman, and Dan Thiel, that seemingly coded around the clock for 18 months to make the 7700 happen. Ray, smiling, was holding an analysis of Tim’s latest run (Figure 7). There it was! We started giggling. The Ct values from these growth curves had lined up pristinely on a plot of Ct versus starting copy number. I wish I had saved that standard plot. Real-time PCR had been detecting small inaccuracies in our independently formulated standards. Real-time PCR was more precise than we ever imagined. Even after amplifying molecules by several orders of magnitude, we were detecting very small differences in starting copy number. This fact was profound and gratifying.

Steve Lombardi, ABI’s Product Manager for DNA sequencing, introduced the book, Crossing the Chasm [23]. It placed the upmost importance on correctly choosing the “headpin” application you knock down first to achieve widespread adoption of a disruptive product. I lobbied hard but unsuccessfully for an HIV kit for pharmaceutical research. An HIV application would have been straightforward to develop, requiring a single or at most a few primer–probe sets, with the downstream potential to create a huge business in diagnostics. However, our de facto headpin application became mRNA gene expression analyses. So, with trepidation I realized we would need to commercialize mRNA TaqMan assays for tens of thousands of genes.

**Figure 8:**
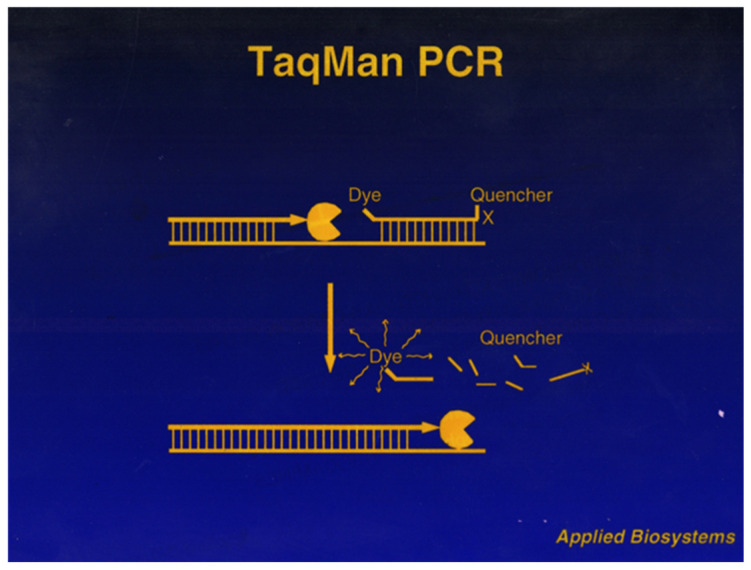
TaqMan probe digestion. 1996 overhead transparency cartoon drawing of a Taq polymerase digesting a 5′-3′ doubly labeled TaqMan probe while annealed to the amplicon.

Now our challenge was to find a synthesis and design strategy for TaqMan probe and primer sets that worked reliably for any target. Ken Livak left DuPont and joined my group in 1994. I soon made him our chemistry leader. Ken developed a universal design motif for TaqMan probes [24]. Counterintuitively, he discovered there was better energy transfer between the reporter (FAM) and quencher (TMR) when attached on opposite ends of the probe (Figure 8). Ken then hired Federico Goodsaid from Abbott who rigorously developed a universal TaqMan PCR cocktail. Using Design Of Experiments (DOE) Federico pinpointed universal biochemical and thermal conditions that married with our algorithm for designing primer–probe sets.

Next, our chemistry team simplified analyses from RNA. In a single tube, we demonstrated TaqMan real-time PCR from mRNA using the components of the EZ rTth RNA PCR Kit that PE commercialized before the merger [25]. This kit was based on reverse transcription activity by Tth DNA polymerase in the presence of manganese. This activity was discovered by David Gelfand’s group [26]. I decided against routinely using this elegant single tube procedure because, at that time, it was incompatible with carry-over contamination control [27]. We stuck with our β-actin DNA kit for system testing and customer installations.

In June 1994, our executives approved my Product Development Plan (PDP). A signed PDP was a key milestone because with it the 7700 would be fully funded through commercialization. They signed it even though its sales forecast page was blank and no headpin application was chosen. I was surprised they did not require our new marketing manager to fill those in. The PDP was fifty pages so perhaps they did not notice.

**Figure 9:**
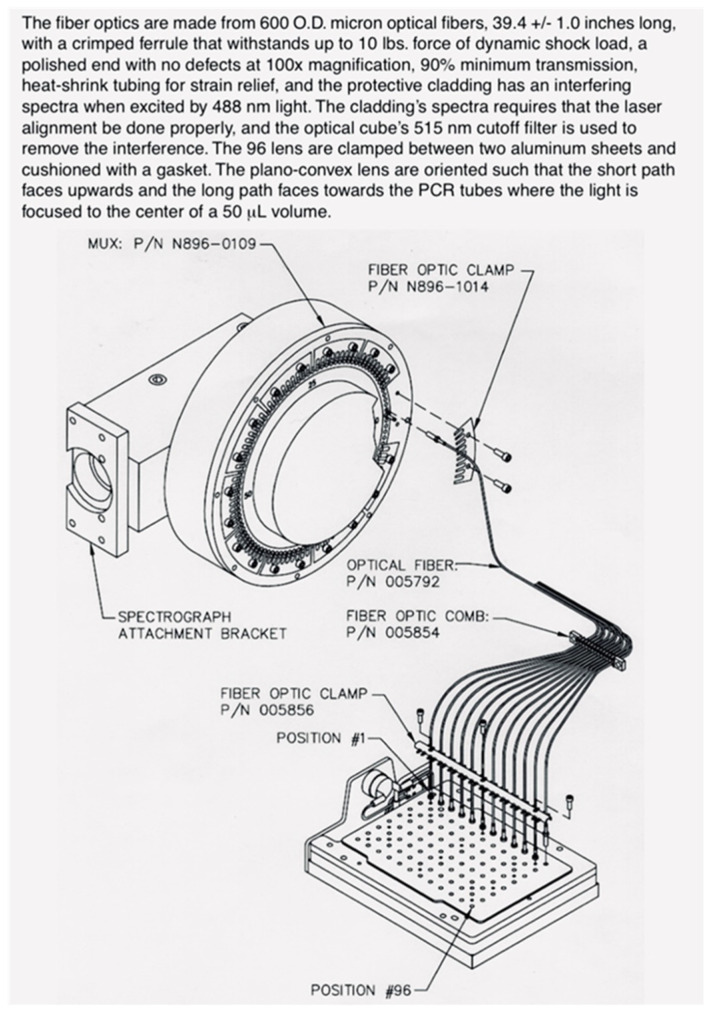
Schematic of fiber-optics, lens assembly, and heated cover. A page from the 7700’s 1996 service manual.

We started building instruments that analyzed 96 samples in microtiter plate format. John Shigeura and Kevin Bodner added a lens above each PCR tube’s optical cap (Figure 6, Right). They routed bundled 96 optical fiber cables from the MUX (Figure 6, Left) in through the 9600 thermal cycler’s heated lid’s enclosure and then fixed the fiber ends above the 96 lenses (Figure 9). Optical caps were molded by Ward Frye from polypropylene but thinner than standard, only 1/100 of an inch. To minimize optical path fluctuations, the lenses did not contact cap centers (Figure 6, Right).

Originally, my engineering team wanted to develop a lightweight low-cost thermal cycler specifically for the 7700. Mike Hunkapiller, then President of the Applied Biosystems Division of PE, voiced concern about the high risk of developing a new thermal cycler from scratch. He advised us to use our commercially available PE 9600 thermal cycler’s hardware. Although the 7700 would be significantly heavier and its cost of goods higher, we would be using proven components. I agreed and I am sure we shipped a more reliable product more quickly.

**Figure 10:**
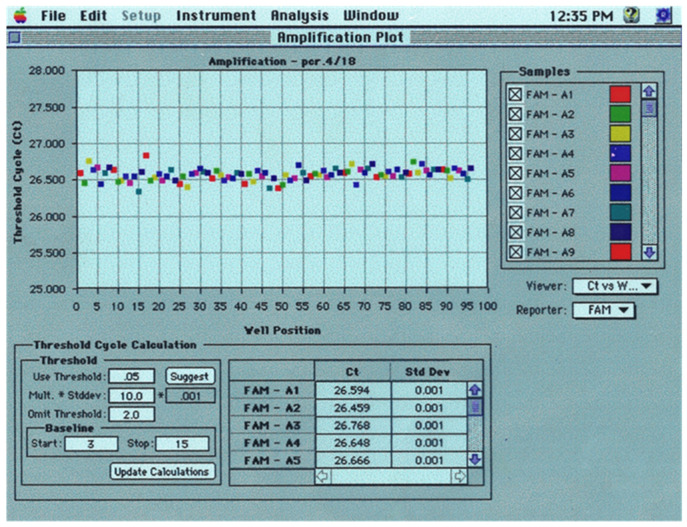
7700 Macintosh screen showing 96 replicates without dropouts. A 1996 presentation overhead showing TaqMan PCR on replicates from the β-Actin DNA Installation Kit with Amplitaq Gold-mediated hot start.

As we manufactured our first 7700s, Bob Grossman, my engineering leader, overhauled the system’s key performance requirement. Our new performance requirement was to resolve two-fold differences in the starting copy number with 99.7% confidence using our β-actin DNA Installation Kit. Our first manufactured 7700 systems showed great promise in meeting this requirement. However, in every run we would see several PCR’s without TaqMan signals. So, to the dismay of Traci Allen, our instrument test leader, I required that, before shipping any unit, it must show 99.7% confidence with two or fewer “dropouts” per 96 reactions on three consecutive runs. Dropouts remained stressful right up to my self-imposed commercial release deadline. Thankfully, in the 11th hour, Paul Wyatt got wind of Roche’s latest Taq polymerase—still in development—called Amplitaq Gold. It was modified with heat labile groups that served to inhibit polymerase activity before the first PCR denaturation step, thereby minimizing non-specific DNA extensions [28]. This new “hot start” method essentially eliminated our dropouts while improving tube-to-tube uniformity in Ct. Figure 10 shows 96 replicates without dropouts and a standard deviation in Ct = 0.085 cycles. This uniformity implied a per-cycle standard deviation in PCR efficiency of less than 0.40%.

**Figure 11:**
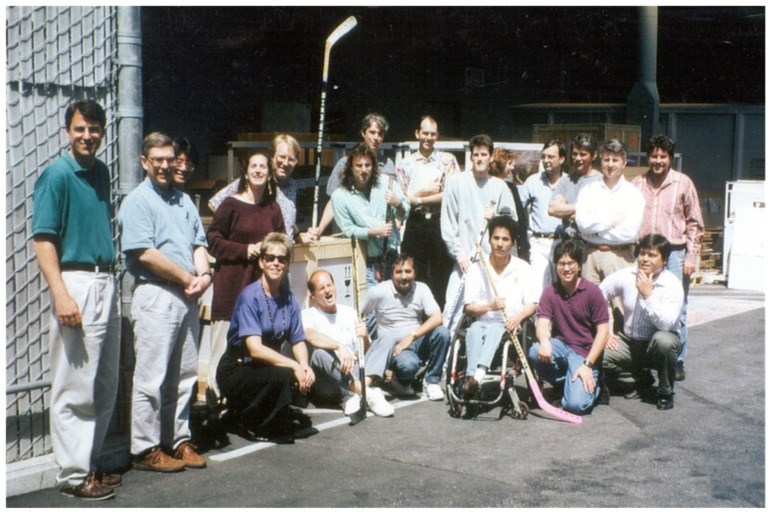
First 7700 prototype shipment in 1995. Sitting left to right: Peggy Wynyard, Kevin Bodner, Mohan Gupta, Jeff Lucas, Bob Grossman, and Mike Lucero. Standing: Bob Ragusa, Ken Livak, John Shigeura, Susan Flood, Jack Jones, 7700 in crate, Dan Thiel, Paul Wyatt, Peter Honebein, Jason Giles, Traci Allen, Ray Lefebvre, Goran Velagic, Gill Ross, and Lincoln McBride.

Russ was curious about the hockey sticks in the 1995 photo of my team at our loading dock shipping our first 7700 (Figure 11). It was going 15 min up HWY 101 to Genentech (recently purchased by Roche), so we could respond rapidly to issues. These hockey sticks remind me of those uncertain times. Having recently recruited Mike Lucero for our marketing manager, I stopped doing marketing on my own. The hockey sticks symbolized the shape of Mike’s first sales forecast. He predicted orders for our $80,000, 350-pound 7700 would start trickling in—its blade. Its long vertical handle symbolized Mike’s hope orders would suddenly shoot through the roof. When our VP of Sales Pat Carroll saw his forecast, he gave these words of encouragement—Mike, you’ll be buried in that 350 pound coffin!

**Figure 12:**
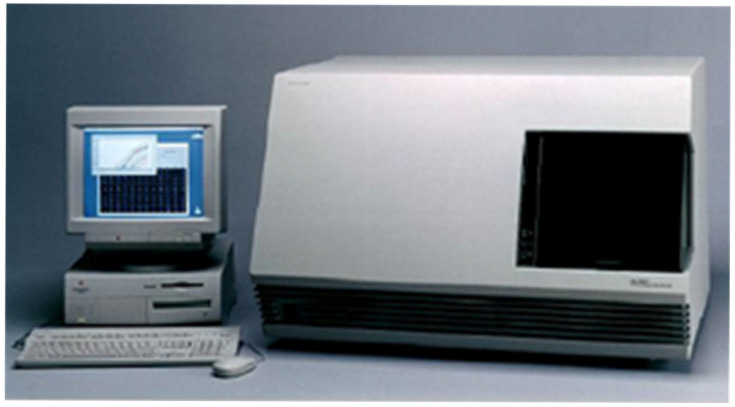
The ABI 7700 Sequence Detection System.

We sold the first seven 7700 systems in the summer of 1996 and 17 more in the fall. In 1997, there were 111 units under warranty. The 7700 (Figure 12) was clearly a big hit. According to Roger Phillips, ABI’s service director, the peak was 2204 units in 2003 under warranty. The 7700 systems’ average mean time between failure (MTBF) was 11.2 months—the best reliability for all ABI first-generation analytical systems. It even bested its successor the 7900, introduced in 2001.

## Russ Higuchi

When we started working on real-time PCR, Roche was developing a quite different automated solution to quantitative PCR. I met with the chief engineer of Tagimenta, Roche’s engineering subsidiary, who was running this project. Their 21-sample instrument achieved the required dynamic range using robotic pipetting for serial diluting HIV samples. PCRs were run with a fixed number of cycles. Amplicon concentrations were then measured by pipetting the completed PCR into a colorimetric probe hybridization reaction that required probe capture and washing on a filter. Starting quantities of virus were estimated with colorimetric OD values after accounting for the level of sample dilution.

I showed him our real-time PCR setup and data that would allow us to avoid all this sample and amplicon manipulation and greatly increase the sample throughput. He said that this technology was very interesting, but he could not tell his engineers about it now, as it would only distract them!

In 1993, we published our second real-time PCR paper describing our digital camera-based system [13] and its quantitative performance. It had adequate precision and the large dynamic range needed for viral load measurement. Ultimately, Roche would not introduce its own real-time PCR instrument (digital camera-based) for diagnostics until 2005, after Abbott that same year, through collaboration with Celera Diagnostics (see below), obtained FDA approval for its HIV test on the m2000rt, an instrument based upon the ABI 7500.

## Lincoln McBride

Junko Stevens transferred to my group in 1995 when the first 7700 systems were starting to hum successfully in-house. One morning as we neared our commercialization deadline, she showed up with a sphygmomanometer. She recorded our blood pressures, and of course mine was high. Presumably her former R&D group did not run quite as hot. A big stressor was my passion to position real-time PCR toward vertical markets like HIV. Roche held tightly onto their PCR rights in human diagnostics. Mike Hunkapiller warned me that if Roche caught wind of us making or supporting HIV assays in any way, even for research purposes, they would cut off our supply of *Taq* Polymerase for DNA Sequencing.

I was certain ABI would not survive in the long term without cracking into diagnostics. I lacked the required persuasive skills, patience, authority, and, at that point, the energy to make that happen. That is when I decided I would leave ABI and be with my two-year old son, Max. I knew my amazing team and product line would continue to do well in the short term without me. In summer 1997, I gave up the fight. I believe it would have been best for all if the 7700 team would have been able to make an HIV research kit then.

## Russ Higuchi

**Figure 13:**
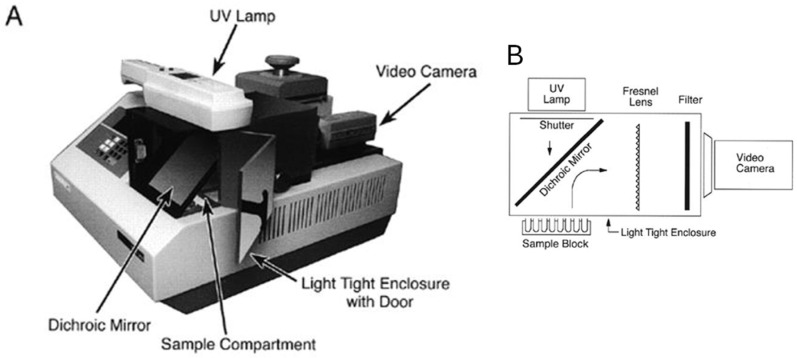
Bob Watson’s prototype, a digital camera-based real-time PCR Instrument with folded optics. (**A**) The optical components of the instrument are contained within a light-tight enclosure positioned over the sample compartment of a Perkin Elmer 9600 thermal cycler. A 300 nm UV lamp shines into the enclosure through a dichroic mirror onto the sample block. (**B**) Fluorescence from the samples is reflected by the dichroic mirror to the rear of the enclosure through a Fresnel lens and interference filter onto a CCD video camera.

Bob Watson continued to work on the camera-based system (Figure 13). He built a system where the long distance between the camera and the thermal cycler was “folded” using mirrors and a Fresnel lens into a metal hood that could fit over the block of a PE9600 thermal cycler [29]. An instrument based on Bob’s design, the GeneAmp 5700, was commercialized in 1999.

In 1993, Kary Mullis received the Nobel Prize in Chemistry for PCR, 10 years after conceiving it. He shared it with Michael Smith, who won for site-directed mutagenesis. Both methods emphasize the importance of synthetic oligonucleotides, which Marvin Caruthers’ group and ABI helped make widely available. Kary Mullis wrote a book about his life, PCR, and the Nobel Prize [30]. A book by Paul Rabinow [31] describes events around the origin of PCR and highlights how teamwork among many scientists and managers at Cetus was crucial in turning the idea into a practical tool.

Realization of the idea most often requires many contributors and their innovations. We remember Alexander Fleming for the discovery of penicillin, but it was years later before Howard Florey, Ernst Chain, and Norman Healy could make enough of it to treat one patient. Ultimately, government, universities, and pharmaceutical companies collaborated to immensely increase production such that soon enough penicillin was being produced to treat all Allied casualties [32].

**Figure 14:**
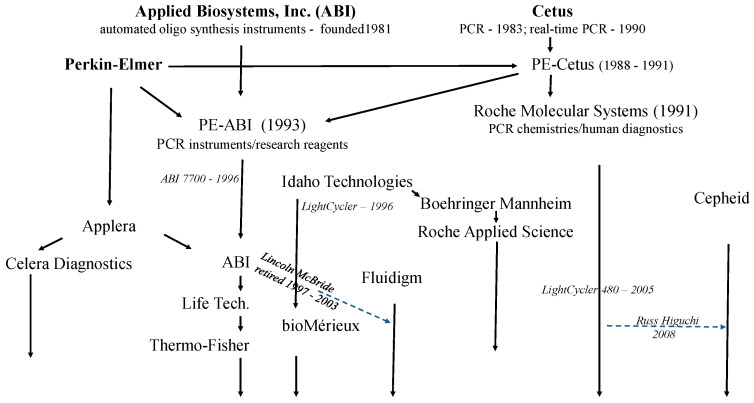
Companies involved with marketing real-time PCR instruments, arranged by relationship and by rough timeline.

The rise of real-time PCR was an endeavor that involved both cooperation and competition between many industrial entities. Lincoln has rightly pointed out how patent license restrictions prevented ABI from pursuing HIV applications for the 7700. I would like to posit, however, that Cetus’, Perkin-Elmers’, and Roche’s licensing policies for PCR did not in general slow the dissemination of PCR technology. This is in fact the conclusion of Fore et al. [33], who did a law and policy case study on this topic. I think this is also borne out by the large number of companies that were licensed for real-time PCR (Figure 14).

The relationships that arose among these companies were exceedingly complex. Even within Roche, different parts of the company had different partners. Boehringer Manheim was acquired by Roche in 1998 but before then had supported work by Idaho Technology, founded by Carl Witwer, who created the LightCycler, a rapid real-time PCR instrument that used glass capillaries and circulating air to heat and cool PCRs and also introduced DNA melt analysis into real-time PCR [34]. The instrument, however, was not suited to high-throughput applications as the loading of the capillaries was difficult to automate. When the companies merged, there were now two separate Roche entities selling different PCR reagents and instruments, Roche Molecular Systems (RMS), where I was, and Roche Applied Science, formed from the reagents group of Boehringer Manheim. Today, the LightCycler name survives but is applied to microtiter plate format, real-time PCR instruments, such as the LightCycler 480, released in 2005. Roche launched its first real-time PCR-based HIV IVD tests on this platform in 2006. This was followed by a fully automated 96-sample system, the COBAS AmpliPrep/COBAS TaqMan System, that included sample preparation, which was a crucial advance for ultimately meeting the throughput challenge of the pandemic.

In 1998, Celera Genomics was founded by Craig Venter and ABI. In 2000, Celera Genomics completed the first working draft of the human genome sequence using a battery of ABI sequencers. In 2002, Tom White and John Sninsky left RMS together with RMS president Kathy Ordonez to start Celera Diagnostics with an initial focus on genetic disease susceptibility. In 2005, Abbott and Celera Diagnostics launched an IVD diagnostics platform used first for HIV viral load measurements, the Abbott m2000 system. This centered on a 96-well, real-time PCR instrument based upon an ABI 7500 and included automated sample prep. Celera Diagnostics is now part of Quest Diagnostics. The founding of Celera Diagnostics is chronicled in [35].

The licensing also created opportunities for scientists with expertise in PCR technology, as seen by the eventual migrations of both of us to different licensees. In 2008, I joined Cepheid, then new to the molecular diagnostics field. Its focus was our point-of-care, sample-in-answer-out, single-use disposable cartridge real-time PCR system. Interestingly, one of its founders was Allen Northrup, who in the mid-90s was a visiting researcher from Lawrence Livermore Labs to Bob Watson’s lab at RMS. There he demonstrated proof of principle of real-time “PCR on a chip”. By the time I joined, Allen was no longer at Cepheid, which had moved on from the microfabricated PCR chip. Today, Cepheid is distinguished by their multiplex real-time PCR, point-of-care, sample-in-answer-out, and single-use disposable cartridge system. This is almost the opposite of a batch-driven, high-throughput microtiter plate format. During the pandemic and after, our alternative proved highly successful in screening patients for critical-use situations like hospital emergency rooms.

## Lincoln McBride

After leaving ABI in the summer of 1997 while keeping an eye on my two young sons, I learned how to swing a hammer. I ended up remodeling three homes largely on my own. The construction process was similar to building first-generation genetic analyzers but was personally more lucrative and most of all gratifying to be nearer to my boys.

One of the reasons Russ invited me to co-author this memoir is because he believes the 7700 system’s architecture remains relevant 30 years later. This longevity is a testament to ABI’s culture and our team’s dedication. Our high-performance group of engineers, scientists, business people, as well as product leads for manufacturing, field service, application support, and product testing worked all together in a no walls office environment—established by ABI’s founders—that fostered mutual learning and rapid problem-solving. A transparent, collaborative culture, built by all ABI employees, was the foundation of our collective and individual success.

In 2003, Mike Lucero coaxed me out of retirement into joining Fluidigm. Their culture made me appreciate how unique ABI was. As ABI’s founding CEO Sam Eletr often explained, “ABI’s mission is to provide the picks and shovels for the biotech gold rush.” This overly humble tagline belied the incredible complexity of the revolutionary products that ABI created and supported. Without doing it yourself, one cannot imagine just how challenging it is to successfully commercialize these first-generation molecular systems. Similarly, it is hard to realize how proud, privileged, and motivated we were to enable our customers’ countless discoveries, which continue to enrich humanity.

By 2003, Applied Biosystems had commercialized mRNA TaqMan assays for most genes, and thus our de facto headpin application for the 7700 was completed. This massive manufacturing effort was championed by Laurent Bellon, now a Cepheid executive.

After a string of confusing name changes, mergers, and acquisitions, the ABI brand and Junko became part of Thermo Fisher Scientific in 2014. Junko remains the steward of ABI real-time PCR. It was arguably ABI’s largest product line in terms of total revenue, and 30 years later real-time PCR is a still a multibillion dollar industry.

The nucleic acid sequences of SARS-CoV-2 (COVID-19), a novel coronavirus, were first published in early January, 2020. US FDA Emergency Use Authorization (EUA) for a real-time PCR test for SARS-CoV-2 (COVID-19) was first granted to the CDC in early February, 2020. This was rapidly followed in March, 2020, by EUA approvals for Roche, Thermo Fisher, Lab Corp, Abbott, and Cepheid real-time PCR-based tests.

The Thermo Fisher/Applied Biosystems TaqPath Assay test for SARS-CoV-2 was designed to provide patient results within four hours of sample receipt. Billions of COVID-19 tests were performed during the pandemic using Thermo Fisher products. We owe Junko and her team’s hard work our thanks. Although from the beginning I yearned for the 7700 to be used to battle HIV, it is far beyond satisfying that in the end Russ’s invention and our group’s 350-pound baby helped ready the world for COVID-19.

## Data Availability

No new data were created or analyzed in this study. Data sharing is not applicable to this article.
